# Comparing methods to measure the dispersion of breathing parameters during exercise testing: A simulation study based on real‐life parameters from patients with dysfunctional breathing

**DOI:** 10.14814/phy2.70233

**Published:** 2025-02-28

**Authors:** Léon Genecand, Cyril Jaksic, Roberto Desponds, Gaëtan Simian, Ivan Guerreiro, Sara Thorens, Marco Altarelli, Isabelle Frésard, Chloé Cantero, Aurélien Bringard, Antoine Beurnier, Pierantonio Laveneziana, David Montani, Anne Bergeron, Frédéric Lador, Pierre‐Olivier Bridevaux

**Affiliations:** ^1^ Service de Pneumologie, Département de Médecine Hôpitaux Universitaires de Genève Genève Switzerland; ^2^ Faculté de Médecine Université de Genève Genève Switzerland; ^3^ Centre de Recherche Clinique Hôpitaux Universitaires de Genève Genève Switzerland; ^4^ Faculté de Mathématique Université de Genève Genève Switzerland; ^5^ Service de Pneumologie Hôpital du Valais, Centre Hospitalier du Valais Romand Sion Switzerland; ^6^ Université Paris‐Saclay, School of Medicine Le Kremlin‐Bicêtre France; ^7^ Institut National de la Santé et de la Recherche Scientifique, Unité Mixte de Recherche S_999 «Pulmonary Hypertension: Pathophysiology and Novel Therapies», HPPIT, Marie Lannelongue Hospital, Le Plessis Robinson France; ^8^ Assistance Publique ‐ Hôpitaux de Paris (AP‐HP), Department of Respiratory and Intensive Care Medicine, Pulmonary Hypertension National Referral Center, FHU André Cournand, ERN‐LUNG, Bicêtre Hospital Le Kremlin‐Bicêtre France; ^9^ AP‐HP, Groupe Hospitalier Universitaire APHP‐Sorbonne Université, Hôpitaux Pitié‐Salpêtrière, Saint‐Antoine et Tenon, Service Des Explorations Fonctionnelles de la Respiration, de l'Exercice et de la Dyspnée (Département R3S) Paris France; ^10^ Sorbonne Université, INSERM, UMRS1158 Neurophysiologie Respiratoire Expérimentale et Clinique Paris France; ^11^ AP‐HP, Groupe Hospitalier Universitaire APHP‐Sorbonne Université, Hôpital Pitié‐Salpêtrière Paris France

**Keywords:** abnormal breathing pattern, cardio‐pulmonary exercise testing, dispersion, dysfunctional breathing, simulations

## Abstract

The dispersion of the tidal volume and of the breathing frequency have been used to diagnose dysfunctional breathing during cardio‐pulmonary exercise testing. No validated methods to objectively describe this dispersion exist. We aimed to validate such a method. We used simulations based on real‐life parameters. Moving standard deviation (MSD) and residuals from locally estimated scatterplot smoothing (LOESS) were evaluated. The precision and the bias of each tested method at rest and during exercise simulations, with and without sighs, were measured. For LOESS, a 2nd degree polynomial was used, and different spans were tested (LOESS_1_, LOESS_0.75_, and LOESS_0.5_). For MSD, different number of points used for the calculation were tested (MSD_7_, MSD_11_, MSD_15_, and MSD19). The LOESS method was globally more precise, had less bias, and was less influenced by the trend as compared to MSD in almost all simulations except for extremely low dispersion combined with extreme trends. LOESS_0.75_ had intermediate bias and precision between LOESS_0.5_ and LOESS_1_ in all simulations. LOESS_0.75_ is a method that combines high precision, low bias, and low influenceability of trends. It could be considered as the method of choice to evaluate the dispersion of breathing parameters during cardiopulmonary exercise testing.

## 1 INTRODUCTION

Many patients have reported persistent symptoms following coronavirus disease 19 (COVID‐19), including dyspnea (Singh et al., 2023). Researchers have hypothesized that dysfunctional breathing (DB) could contribute to some of these persistent symptoms (Beurnier et al., 2023; Fresard et al., 2022; Genecand et al., 2023; Guerreiro et al., 2023; Motiejunaite et al., 2021). DB is not a new phenomenon discovered during the COVID‐19 pandemic. It has been observed before the pandemic, occurring in patients with respiratory or cardiovascular diseases, as well as in those with anxiety disorders or in patients without any associated comorbidities (Boulding et al., 2016). DB is typically defined by the presence of compatible symptoms, an abnormal breathing pattern, and the exclusion of cardio‐pulmonary or metabolic causes fully explaining these symptoms (Boulding et al., 2016). Normal breathing is usually under unconscious control and generally exhibits a low dispersion of breathing parameters and a low number of sighs (Tobin et al., 1983a). The breathing can become dysregulated, characterized by excessive sighing, irregular breathing frequency (BF), and high variations of tidal volume (VT) (Abelson et al., 2001; Beurnier et al., 2023; Fresard et al., 2022; Genecand et al., 2023; Prys‐Picard et al., 2006). This is believed to potentially induce symptoms through various mechanisms such as inefficient ventilation and poor subjective awareness of breathing (Boulding et al., 2016).

**FIGURE 1:**
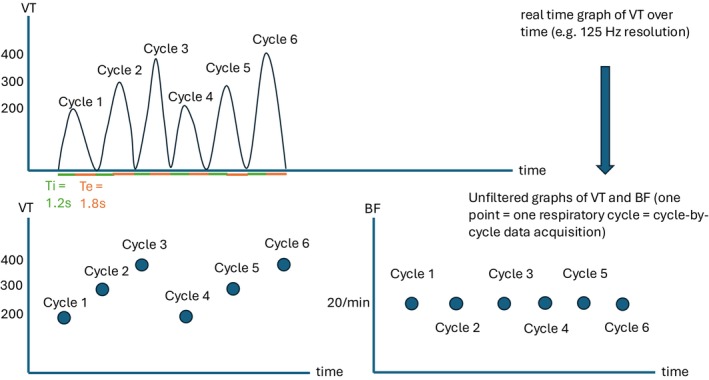
From real‐time volume‐time plots to unfiltered graphs. The high‐resolution volume‐time curve represents the continuous volume‐over‐time curve. The VT unfiltered graph represents the absolute tidal volume of every respiratory cycle from the patient. The BF unfiltered graph represent the BF of each respiratory cycle which is calculated as 60/(time of the respiratory cycle in seconds). In the present example, the time from the respiratory cycle is 3 seconds (1.2 + 1.8 s) and is constant for the 6 cycles. Therefore, the BF is 20/min for each respiratory cycle.

**FIGURE 2:**
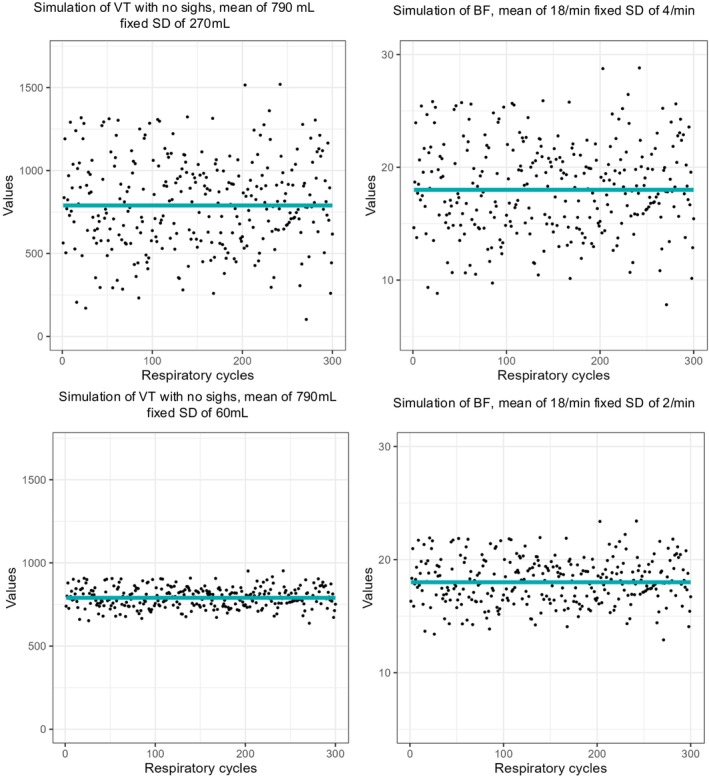
Illustration of the concept of dispersion of breathing parameters at rest using an unfiltered cycle‐by‐cycle graph. In this figure, we simulated two different dispersions for both VT and BF at rest (no trend). Each point on the graph represents one respiratory cycle. The BF value is, for every cycle, calculated as 1 divided by the addition of the inspiratory and expiratory times measured in minutes. On the left‐hand side, the two VT simulations are shown with a mean of 790 mL and a SD of 270 mL (upper graph) or of 60 mL (lower graph). One can observe on the upper graph, the large variability of VT with a mean VT of 790 mL and 95% of the VT points contained between a VT of 261 and 1319 mL as compared to the example on the lower graph with a mean VT of 790 mL with 95% of the VT points contained between a VT of 672 and 908 mL. On the right‐hand side, the two BF simulations are shown with a mean of 18/min, a SD of 4/min (upper graph), and of 2/min (lower graph). One can observe on the upper graph a more irregular breathing with a mean breathing of 18/min and 95% of the BF points contained between 10.2 to 25.8 breaths/min compared to the example on the lower graph with a mean BF of 18/min and 95% of the BF points contained between 14.1 and 21.9/min (i.e., the breathing is more regular). Similar reasoning can be applied when interpreting dispersion around the trended parameters during exercise.

Cardiopulmonary exercise testing (CPET) is a valuable tool that allows to explore the cause of dyspnea and exercise limitation including abnormal breathing patterns (Laveneziana et al., 2021; Watson et al., 2021). For these reasons, CPET has been extensively used to evaluate dyspnea after COVID‐19 and to diagnose DB (Durstenfeld et al., 2022). Using a cycle‐by‐cycle analysis, one can obtain for every respiratory cycle a VT and a BF value. Figure 1 illustrates how unfiltered cycle‐by‐cycle graphs are created from high‐resolution data volume over time graph. As shown in Figure 2, the dispersion of VT describes how variable VT is and the dispersion of BF describes how irregular the breathing is. Unfiltered cycle‐by‐cycle graphs of VT and BF during rest and exercise have been used to visually quantify erratic breathing and sighs and to diagnose DB (Boulding et al., 2016; Fresard et al., 2022; Genecand et al., 2023; Ionescu et al., 2020). However, visual analyses have been met with skepticism due to their subjective nature. Therefore, the use of an objective method to describe the dispersion is required. This could enable researchers to develop normative values for the dispersion of breathing, search for associations between objective markers of DB and symptoms of DB, evaluate new prognostic markers, and find diagnostic cutoffs for abnormal breathing patterns. Few objective methods have been tested to describe the dispersion of breathing parameters during exercise (Brutsche et al., 2023), and the optimal method to describe the dispersion of VT and BF remains unknown. The main challenge to evaluate breathing dispersion during CPET lies in the trend of the data. Indeed, both VT and BF increase during exercise, but their increase is frequently non‐linear, and the trend varies between individuals. Without trend (e.g., at rest), simple methods such as standard deviation (SD) would provide a perfectly acceptable measurement of data's dispersion around their mean. These methods have been used for decades to describe the dispersion of breathing parameters at rest (Tobin et al., 1983a, 1983b). However, the trend of breathing parameters during exercise makes such methods inappropriate. The ideal objective method to describe dispersion of breathing parameters should accurately and precisely describe the amplitude of VT and BF dispersion without being affected by the shape and steepness of the data's trend. By trend, we refer to a consistent pattern in which data values tend to increase or decrease over time.

One problem for researchers comparing the performance of different methods when no gold standard exists is the formal impossibility to determine the best method (Morris et al., 2019). As a result, real‐life data are not appropriate for method development. As a first step, it is common to compare the performance of methods on simulated data, which allows to know the true value of all parameters and thus have precise estimate of the methods' performance (Austin, 2011; Malehi et al., 2015; Spiess & Neumeyer, 2010). For this reason, we tested two methods on simulated data, with parameters derived from real data, to identify an appropriate method for estimating the true dispersion of breathing parameters during exercise. In the present article, the true dispersion refers to the dispersion of data around the trended parameter. Our aim was to evaluate the accuracy and precision of two methods for assessing the dispersion of VT and BF: (1) the moving standard deviation (MSD) and (2) the locally estimated scatterplot smoothing (LOESS).

## 2 MATERIALS AND METHODS

We considered that the ideal method for the measurement of the dispersion of VT and BF should:

1. Describe the dispersion of both VT and BF with a low bias and a high precision.
2. Be unaffected by the trend of the measured data, meaning that the trend should not compromise the method's ability to describe the true dispersion of the breathing parameters.

The true dispersion was defined as the standard deviation (SD) around the mean of simulated data before applying the trend.

We defined the bias as the difference between the mean value of the dispersion of either VT or BF obtained with the tested method and the true dispersion (set by the simulated SD). The bias represents the capacity of the tested method to yield, on average, a value close to the true value. We defined the precision as the SD of the dispersion of either VT or BF as measured by the tested method. The precision reflects the capacity of the tested method to consistently yield the same value.

### 2.1 Real‐life data used for the simulations

Parameters set for the simulated data were based on a case series of 48 patients diagnosed with DB after COVID‐19 (Genecand et al., 2023). The mean (SD) age of the participants was 48.5 (15.0) years, and 33 individuals (68.8%) were women. This case series included patients diagnosed with hyperventilation (HVS), erratic breathing, or periodic deep sighs (EB/PDS) or a mixed of these patterns. Briefly, we included patients, aged 15 or older, diagnosed with DB at our long COVID outpatient clinic. DB was diagnosed based on the following combined approach: the presence of compatible symptoms, including significant dyspnea defined by a modified Medical Research Council (mMRC) score ≥1, combined with other symptoms related to DB and the presence of an abnormal breathing pattern (HVS, EB/PDS, or a mixed pattern) during CPET evaluation, after exclusion of pathologies that could fully explain these symptoms. The complete description of the study population and clinical setting are detailed elsewhere (Genecand et al., 2023).

### 2.2 Principle of the simulations

**FIGURE 3:**
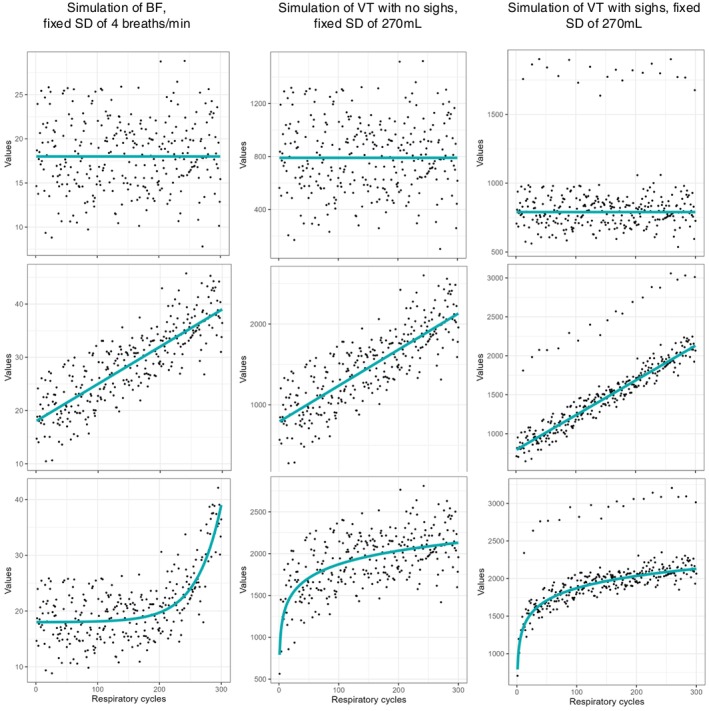
Example of simulations. Simulations using a SD of 270 mL for VT with a normal distribution of data, a SD of 4/min for BF with a normal distribution of data and a SD of 270 mL for VT with sighs. For each simulation, a trend was then applied (linear and logarithmic for VT, exponential for BF.

First, the VT and BF data were simulated with no trend (flat), a fixed mean value, and a known dispersion (set by the SD). This SD represents, by definition, the “true” dispersion of the evaluated breathing parameter and can be readily calculated by computing the SD of the flat trend. Different trends were then applied to those data to simulate exercise measurements as shown in Figure 3. Finally, the tested methodologies were compared to the true dispersion of the data. Bias and precision were determined in all situations.

### 2.3 Determination of the trend applied to the simulated data for VT and BF using real‐life data

Typical trends for VT and BF are usually represented by a logarithmic trend and an exponential trend, respectively. However, in our case series, the trend of VT ranged from a markedly curved logarithmic to an almost linear increase during exercise (Genecand et al., 2023). Similarly, the trend of BF ranged from a markedly curved exponential to an almost linear increase during exercise . We simulated the two most extreme trends for each case (VT and BF). For VT, a markedly curved logarithm and a linear increase were simulated. For BF, a markedly curved exponential and a linear increase were simulated. If statistical methods remained unaffected by the extreme trends, we assumed that they should be similarly robust for all trends falling between the two extremes.

### 2.4 Determination of the range of VT and BF in the simulated data

Based on our previous case series (Genecand et al., 2023), we established the following parameters for the simulations: the mean resting VT was set at 790 mL, with an average end‐of‐effort VT of 2130 mL. The mean resting BF was set at 18 breaths per minute (min), with an average end‐of‐effort BF of 39/min. The number of respiratory cycles during the whole test was set at 300, which represented the average number of VT and BF data obtained by the breath‐by‐breath analysis during CPET in our case series (Genecand et al., 2023). We plotted VT and BF over the number of respiratory cycles. This is a proxy of time matching the nature of data collected and analyzed during routine effort test.

### 2.5 Determination of the amplitude of the dispersion in the simulated data for VT and BF

We calculated the moving standard deviation of 7 points (MSD7) of VT and BF at rest and during exercise in our case series of patients with DB (Genecand et al., 2023). The aim was not to define the “true” dispersion of VT and BF at rest and during exercise, as the optimal method to measure it remains unknown. However, we considered that MSD7 should give a reasonable estimation of standard deviation to simulate plausible values. The mean MSD7 of VT in our 48 patients at rest, during moderate exercise (5th minute of exercise), and the complete exercise were respectively 270, 280, and 250 mL. The mean MSD7 of BF in our 48 patients at rest, during moderate exercise and the complete exercise were, respectively, 4.4/min, 4.0/min, and 3.9/min. Since the mean MSD7 value for both VT and BF remained very stable, we decided to use similar values for the simulations at rest (non‐trended values) and during exercise (trended values). Based on the MSD7 of our case series, we simulated data with mean dispersions for VT and BF at SD = 270 mL and SD = 4/min, respectively. Based on our case series, we simulated data corresponding to the extreme of VT and BF dispersion that we observed. For VT, a SD of 60 and 500 mL were used, corresponding to very little dispersion of VT and very high dispersion of VT, respectively. For BF, a SD of 2 and 6 breaths/min were used, corresponding to very regular breathing and very irregular breathing, respectively.

### 2.6 Simulations of pure erratic breathing

We defined “pure erratic breathing” as a large dispersion of breathing parameters without regular outliers (e.g., sighs for VT). By “large,” we refer to simulated parameters based on the most extreme observed data in the patients from our cohort (Genecand et al., 2023). For these simulations, we used a normal distribution of the breathing parameter (VT and BF), as this was the most typical distribution observed in our previous case series for patients considered to have pure erratic breathing and no obvious sighs.

To simulate VT, we generated 10,000 simulations of 300 randomly and normally distributed data with a mean of 790 mL and a SD of 270 mL with no trend in the data (i.e., flat). A trend was then applied to mimic VT evolution during exercise (linear and logarithmic trends). A targeted mean VT of 2130 mL at the end of the exercise was used. This process was replicated using a SD of 60 mL and a SD of 500 mL and keeping the same mean resting VT and mean end of exercise VT.

To simulate BF, we generated 10,000 simulations of 300 randomly and normally distributed data with a mean of 18 breaths/min and a SD of VT of 4 breaths/min with no trend in the data. A trend was then applied to mimic BF evolution during exercise (linear and exponential trends). A targeted mean BF of 39/min at the end of the exercise was used (mean end of exercise BF as observed in our case series). This process was replicated using a SD of 2 breaths/min and a SD of 6 breaths/min and keeping the same mean resting BF and mean end of exercise BF.

### 2.7 Simulations mimicking sighs

For the simulations of sighs, we drew values from a normal distribution centered on 3500 mL with a SD of 200 mL. Values from the original simulated dataset (from a normal distribution of mean VT value 790 mL, SD 270 mL) were randomly replaced by those sighs at an average interval of 15 cycles (SD = 3), but not closer than 4 cycles apart. After the data were replaced, the overall SD of the flat data was adjusted to correspond to 270 mL. Trends (linear and logarithmic) were applied after sighs were inserted into the data, meaning that trends were also applied to the sighs. A targeted mean VT of 2130 mL at the end of the exercise was used (mean end of exercise VT as observed in our case series). This was replicated using a fixed SD of 60 and of 500 mL and keeping the same mean resting VT and mean end of exercise VT.

We gave a summary of the simulated parameters in Table 1.

**TABLE 1:** Summary of the simulations.

	Simulation of BF	Simulations of VT	Simulation of VT with sighs
Mean simulated parameter at rest	18/min	790 mL	790 mL
Mean simulated parameter at peak exercise	39/min	2130 mL	2130 mL
Simulated dispersion. Expressed in standard deviation	2/min	60 mL	60 mL
	4/min	270 mL	270 mL
	6/min	500 mL	500 mL
Simulated distribution	Normal distribution of data	Normal distribution of data	Normal distribution of data, random replacement of points with sighs keeping the overall dispersion fixed.
Initial trend for simulation	Flat	Flat	Flat
Type of trends applied to test the methods	Linear	Linear	Linear
	Exponential (e^40^)	Logarithmic	Logarithmic
Number of iterations for each scenario	10′000	10′000	10′000

*Note*: Summary table of all the simulated data.

Abbreviations: BF, breathing frequency; VT, tidal volume.

### 2.8 Methods evaluated

We selected the MSD and the LOESS methods because they are non‐parametric, meaning they do not make assumptions about the type or form of trends. Thus, they have the potential to fit different trends.

**FIGURE 4:**
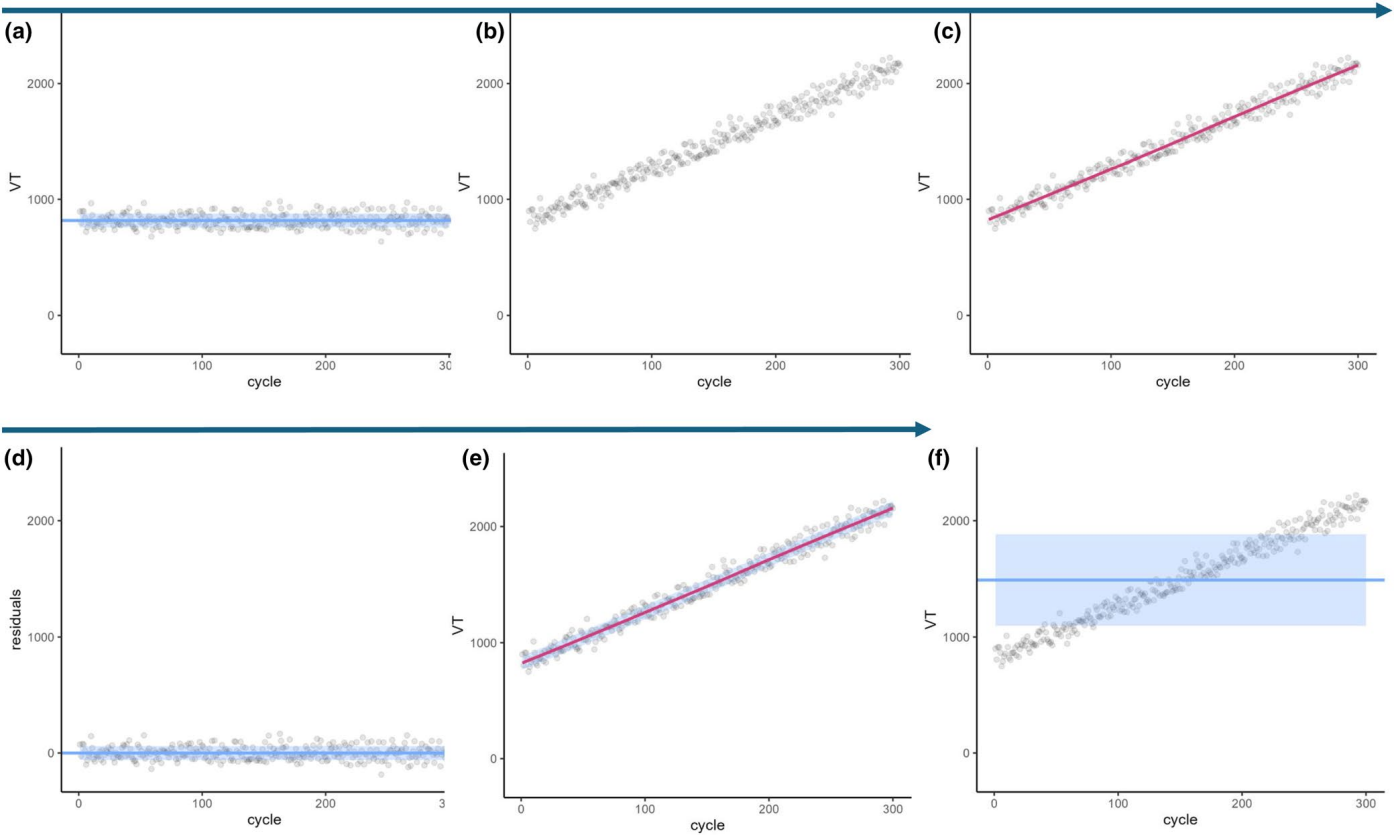
Applications of the LOESS_0.75_ method. Panel (a) represents a simulated data set with a mean of 790 mL (horizonal bar) and a SD of 60 mL (blue bars). In panel (b) a linear trend is applied to the data set, which now goes from 790 mL (resting volume) to 2100 mL (end of exercise simulated volume). (c) The LOESS_0.75_ method (non‐parametric regression method) is estimated fitting a curve in the trended dataset (redline). From this curve, the residuals are calculated, which are the vertical differences between each point and the curve. Panel (d) shows the calculated residuals, which are, as expected, centered on 0 with a SD closely representing the SD from the dataset before the trend was applied as illustrated in panel a. In panel (e) the SD as calculated on the residuals is represented around the curve of the LOESS. Therefore, the SD as calculated on the residuals, represents the SD around the trended parameter and can be used to estimate the dispersion around the trend. For simplicity and graphical representation this is shown using a linear trend, but a similar result would be obtained with a logarithmic trend. The panel (f) represents the SD simply calculated on the dataset with the trends (SD_trend_) showing that the SD in this situation do not represent only the dispersion of the dataset but is largely influenced by the trend.

LOESS is a non‐parametric regression method based on the moving average and polynomial regression analysis. It allows to model non‐linear relationships. Using this method allows to estimate the trend and subsequently estimate the SD of the data by calculating the SD of the residuals. A graphical example of how the LOESS operates is given in Figure 4. The span is the parameter of the LOESS that controls the degree of smoothing with higher values leading to greater smoothing. A default span of 0.75 is, for example, suggested in R software. The choice of the span is a tradeoff between poor fit of the data and overfitting of the data. For the simulations, we chose to test LOESS with a span of 0.5 (LOESS_0.5_), 0.75 (LOESS_0.75_), and 1 (LOESS_1_).

Moving standard deviation (MSD) is a method that calculates the SD for a given number of “n” consecutive data points (e.g., 7 points), then repeating the calculation by shifting one point at a time. The MSDn represents the average SD calculated by averaging all SDn values. To define a good “n” to be used, two elements should be weighted. The SD is biased when very low numbers of data are used for its measurement, leading to an underestimation of the true value of the SD. The smaller the numbers of included variables, the higher the bias. For values of 3, 5, and 7, the SD will underestimate the true value of the SD by an average of 11%, 6%, and 3% as can be found through simulations (cf R code 1, Appendix S1). On the other hand, larger numbers of variables used in the MSD will result in a measurement being inexorably influenced by the trend of the measured data leading to an overestimation of the parameters when a trend is present. Therefore, “n” should be chosen to be not too small (to not consistently underestimate the true value) and not too large (to not consistently overestimate the true value due to the trend's influence). For the simulations, we have chosen to test values for “n” of 7, 11, 15, and 19.

Statistical software R (version 4.2.2) was used. The code used in R for the simulations is joined in Appendix S1. The data availability statement concerning the patients included in the case series is available elsewhere (Genecand et al., 2023). The code used for the simulations is free of use. Nevertheless, the authors require that their work be cited if this method is used to measure the dispersion of breathing parameters either for research or in CPET software used for clinical purposes.

## 3 RESULTS

Figure 3 shows examples of simulated data and the different evaluated trends for VT (normally distributed, SD 270 mL with and without sighs) and BF (normally distributed, SD 4/min). The top section of the figure displays data simulated without a trend. The middle section shows a linear trend, while the bottom section presents the simulated VT data with a logarithmic trend and the BF data with an exponential trend.

**FIGURE 5:**
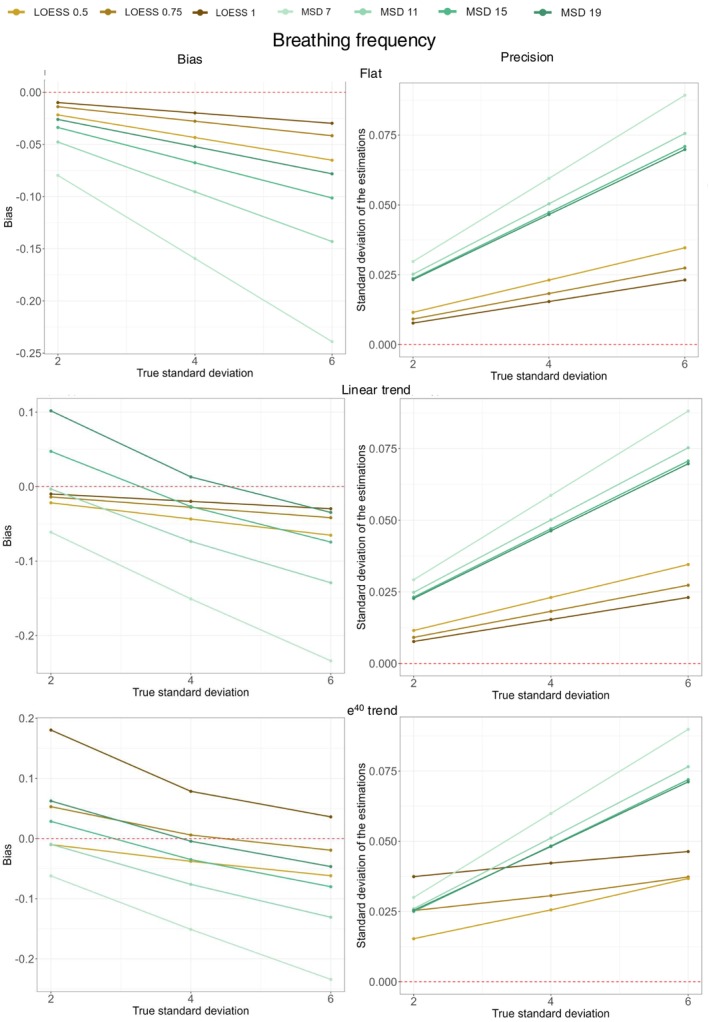
Bias and precision for the simulations of BF. The bias (left‐hand side of the figure) or precision (right‐hand side of the figure) is represented on the *y*‐axis. They are plotted against the true SD on the *x*‐axis (with SD simulated SD of 2/min, 4/min, and 6/min). Each graphic represents either the bias or the precision according to one type of trend (from top to bottom: flat, linear, and exponential trend). The red line represents no bias (left‐hand side) or perfect precision (right‐hand side). The bias is calculated as the difference between the mean value of the dispersion of BF obtained with the tested method and the true dispersion (set by the simulated SD). The bias represents the capacity of the tested method to yield, on average, a value close to the true value. The precision was defined as the SD of the dispersion of BF as measured by the tested method. The precision reflects the capacity of the tested method to consistently yield the same value. All the values represented in Figure 5 can be recalculated from Table 2.

Table 2 shows the results for the simulations of BF following a normal distribution and with various trends. The three simulated scenarios, with SD of 2, 4, and 6, are shown. The MSD and the LOESS methods were evaluated on simulated data across three conditions: no trend (flat), a linear trend, and an exponential trend. The bias was calculated as the difference between the true SD and the mean SD estimated by each method across 10,000 simulations (i.e., true SD – mean estimated SD). The precision was calculated as the SD of the estimated parameter across these 10,000 simulations. For example, in Table 2, in the scenario with a true SD of 2 and a linear trend, the LOESS_0.50_ method yields an estimate of 1.98 (±0.01). Thus, the bias of this method is −0.02 (1.98–2), and the precision (1 SD) is 0.01. Since the dispersion estimation provided by the LOESS followed a normal distribution, 95% of the observations lie within ±1.96 SD of the mean value. In this specific scenario, this implies that 95% of the estimations fall between 1.96 and 2.00. Figure 5 visually depicts the bias (left side, y‐axis) and precision (right side, *y*‐axis) for each method. The three conditions—no trend (flat), linear trend, and exponential (e^40^) trend—are displayed from top to bottom. The methods are shown with a color gradient: from dark brown to yellow‐brown for LOESS and from light green to dark green for MSD. The *x*‐axis of each figure shows the three simulated SD (2, 4, and 6), and the red line represents zero bias (left side) and perfect precision (right side).

**TABLE 2:** Simulations of breathing frequency.

Methods	Flat (no trend)	Linear trend	Exponential trend
SD of 2			
SD	2 (±0)	6.39 (±0.11)	5.12 (±0.11)
LOESS_1_	1.99 (±0.01)	1.99 (±0.01)	2.18 (±0.04)
LOESS_0.75_	1.99 (±0.01)	1.99 (±0.01)	2.05 (±0.03)
LOESS_0.50_	1.98 (±0.01)	1.98 (±0.01)	1.99 (±0.02)
MSD_7_	1.92 (±0.03)	1.94 (±0.03)	1.94 (±0.03)
MSD_11_	1.95 (±0.03)	2 (±0.02)	1.99 (±0.03)
MSD_15_	1.97 (±0.02)	2.05 (±0.02)	2.03 (±0.03)
MSD_19_	1.97 (±0.02)	2.1 (±0.02)	2.06 (±0.03)
SD of 4			
SD	4 (±0)	7.27 (±0.19)	6.18 (±0.18)
LOESS_1_	3.98 (±0.02)	3.98 (±0.02)	4.08 (±0.04)
LOESS_0.75_	3.97 (±0.02)	3.97 (±0.02)	4.01 (±0.03)
LOESS_0.50_	3.96 (±0.02)	3.96 (±0.02)	3.96 (±0.03)
MSD_7_	3.84 (±0.06)	3.85 (±0.06)	3.85 (±0.06)
MSD_11_	3.9 (±0.05)	3.93 (±0.05)	3.92 (±0.05)
MSD_15_	3.93 (±0.05)	3.97 (±0.05)	3.97 (±0.05)
MSD_19_	3.95 (±0.05)	4.01 (±0.05)	4 (±0.05)
SD of 6			
SD	6 (±0)	8.54 (±0.25)	7.63 (±0.21)
LOESS_1_	5.97 (±0.02)	5.97 (±0.02)	6.04 (±0.05)
LOESS_0.75_	5.96 (±0.03)	5.96 (±0.03)	5.98 (±0.04)
LOESS_0.50_	5.93 (±0.03)	5.93 (±0.03)	5.94 (±0.04)
MSD_7_	5.76 (±0.09)	5.77 (±0.09)	5.77 (±0.09)
MSD_11_	5.86 (±0.08)	5.87 (±0.08)	5.87 (±0.08)
MSD_15_	5.9 (±0.07)	5.93 (±0.07)	5.92 (±0.07)
MSD_19_	5.92 (±0.07)	5.97 (±0.07)	5.95 (±0.07)

*Note*: Simulations of BF with a true standard deviation of 2/min, 4/min, and 6/min. The data were simulated randomly with a normal distribution.

Abbreviations: LOESS_n_, standard deviation of residuals from locally estimated scatterplot smoothing with a span of n; MSD_n_, moving standard deviation using “n” data for the calculation of individual SD.

In the bias graphs (left side), we observe that LOESS generally shows low bias for data without a trend (flat, top‐left panel), with the largest bias being −0.07 for the LOESS_0.5_ estimate at a simulated SD of 6. In contrast, the MSD method shows a higher overall bias, peaking at −0.24 with the MSD7 method for a simulated SD of 6. These values are detailed numerically in Table 2. Regarding precision, for data without a trend (top‐right panel), similar patterns emerge, with MSD showing lower precision than LOESS. The lowest precision is seen with MSD7 in the SD of 6 scenario, where precision is 0.09.

The remaining data of Table 2 and Figure 5 illustrate the bias and precision of each method across all simulated scenarios and trends.

**FIGURE 6:**
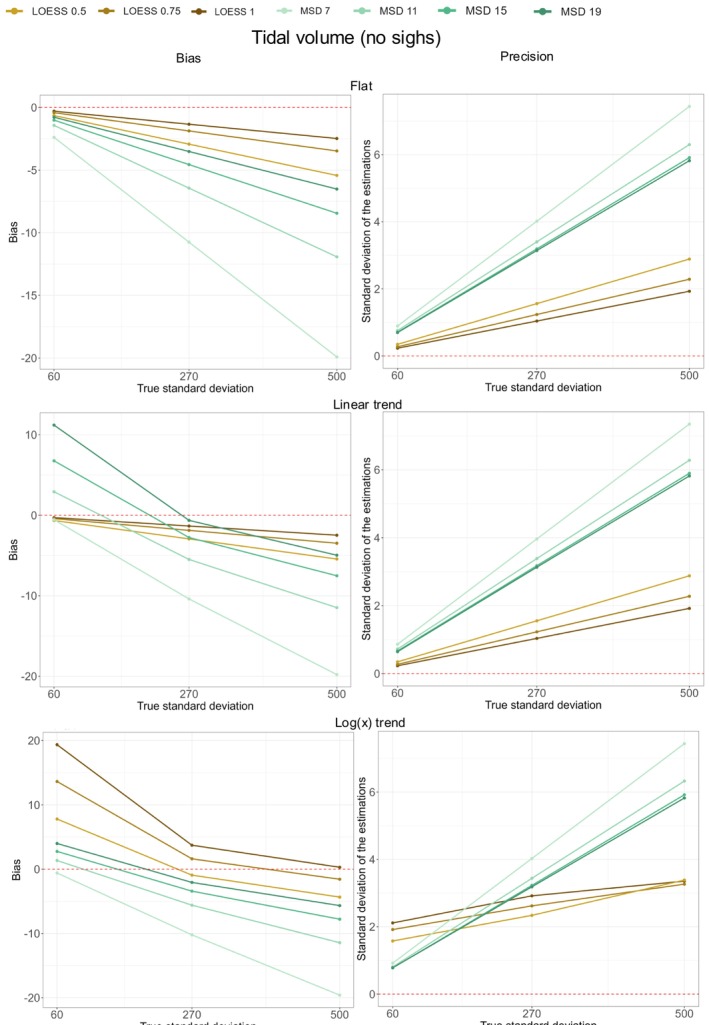
Bias and precision for the simulations of VT without sighs. The bias (left‐hand side of the figure) or precision (right‐hand side of the figure) is represented on the *y*‐axis. They are plotted against the true SD on the *x*‐axis (with simulated SD of 60, 270, and 500 mL. Each graphic represents either the bias or the precision according to one type of trend (from top to bottom: flat, linear and logarithmic trend). The red line represents no bias (left‐hand side) or perfect precision (right‐hand side). The bias is calculated as the difference between the mean value of the dispersion of VT obtained with the tested method and the true dispersion (set by the simulated SD). The bias represents the capacity of the tested method to yield, on average, a value close to the true value. The precision was defined as the SD of the dispersion of VT as measured by the tested method. The precision reflects the capacity of the tested method to consistently yield the same value. All the values represented in Figure 6 can be recalculated from Table 3.

Table 3 shows the results for the simulations of VT following a normal distribution and with various trends. Figure 6 shows the corresponding bias and precision for the two methods. The analysis approach is similar to the one described above for BF.

**TABLE 3:** Simulations of tidal volume without sighs.

Methods	Flat (no trend)	Linear trend	Logarithmic trend
SD of 60 (no sighs)			
SD	60 (−)	392.1 (±3.4)	234.7 (±3.4)
LOESS_1_	59.7 (±0.2)	59.7 (±0.2)	79.4 (±2.1)
LOESS_0.75_	59.6 (±0.3)	59.6 (±0.3)	73.6 (±1.9)
LOESS_0.50_	59.4 (±0.4)	59.4 (±0.4)	67.8 (±1.6)
MSD_7_	57.6 (±0.9)	59.5 (±0.9)	59.4 (±0.9)
MSD_11_	58.6 (±0.8)	62.9 (±0.7)	61.4 (±0.8)
MSD_15_	59.0 (±0.7)	66.8 (±0.7)	62.8 (±0.8)
MSD_19_	59.2 (±0.7)	71.2 (±0.7)	64.0 (±0.8)
SD of 270 (no sighs)			
SD	270 (−)	472.2 (±12.8)	352.6 (±10.0)
LOESS_1_	268.7 (±1.0)	268.7 (±1.0)	273.7 (±2.9)
LOESS_0.75_	268.1 (±1.2)	268.1 (±1.2)	271.6 (±2.6)
LOESS_0.50_	267.1 (±1.6)	267.1 (±1.6)	269.1 (±2.3)
MSD_7_	259.3 (±4.0)	259.6 (±4.0)	259.8 (±4.0)
MSD_11_	263.6 (±3.4)	264.5 (±3.4)	264.4 (±3.4)
MSD_15_	265.4 (±3.2)	267.2 (±3.2)	266.6 (±3.2)
MSD_19_	266.5 (±3.1)	269.4 (±3.1)	267.9 (±3.2)
SD of 500 (no sighs)			
SD	500 (−)	632.5 (±17.8)	549.1 (±11.9)
LOESS_1_	497.5 (±1.9)	497.5 (±1.9)	500.3 (±3.4)
LOESS_0.75_	496.5 (±2.3)	496.5 (±2.3)	498.4 (±3.3)
LOESS_0.50_	494.6 (±2.9)	494.6 (±2.9)	495.7 (±3.4)
MSD_7_	480.1 (±7.4)	480.2 (±7.4)	480.4 (±7.4)
MSD_11_	488.1 (±6.3)	488.5 (±6.3)	488.6 (±6.3)
MSD_15_	491.6 (±5.9)	492.5 (±5.9)	492.2 (±5.9)
MSD_19_	493.5 (±5.8)	495.0 (±5.8)	494.3 (±5.8)

*Note*: Simulations of VT with a true standard deviation of 60 mL, 270 mL and 500 mL. The data were simulated randomly with a normal distribution (no sighs).

Abbreviations: LOESS_n_, locally estimated scatterplot smoothing with a span of n; MSD_n_, moving standard deviation using “n” data for the calculation of individual SD.

**FIGURE 7:**
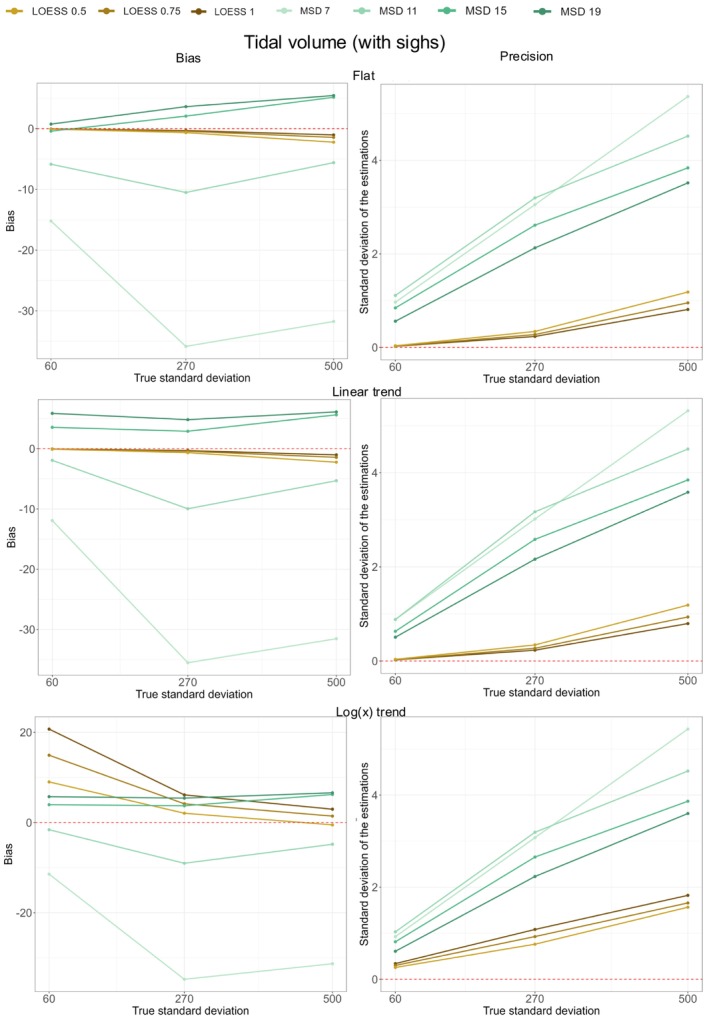
Bias and precision for the simulations of VT with sighs. The bias (left‐hand side of the figure) or precision (right‐hand side of the figure) is represented on the *y*‐axis. They are plotted against the true SD on the *x*‐axis (with simulated SD of 60, 270, and 500 mL. Each graphic represents either the bias or the precision according to one type of trend (from top to bottom: flat, linear and logarithmic trend). The red line represents no bias (left‐hand side) or perfect precision (right‐hand side). the bias is calculated as the difference between the mean value of the dispersion of VT obtained with the tested method and the true dispersion (set by the simulated SD). The bias represents the capacity of the tested method to yield, on average, a value close to the true value. The precision was defined as the SD of the dispersion of VT as measured by the tested method. The precision reflects the capacity of the tested method to consistently yield the same value. All the values represented in Figure 7 can be recalculated from Table 4.

Table 4 shows the results for the simulation of VT following a normal distribution with sighs added to it and with various trends applied. Figure 7 shows the corresponding bias and precision for the two methods. The analysis approach is similar to the one described above for BF.

**TABLE 4:** Simulations of tidal volume with sighs.

Methods	Flat (no trend)	Linear trend	Logarithmique trend
SD of 60 (with sighs)			
SD	60 (±0)	392.7 (±0.9)	235.9 (±0.8)
LOESS_1_	60.0 (±0.0)	60.0 (±0.02)	80.7 (±0.3)
LOESS_0.75_	60.0 (±0.0)	60.0 (±0.03)	74.9 (±0.3)
LOESS_0.50_	59.9 (±0.0)	59.9 (±0.04)	69 (±0.3)
MSD_7_	44.8 (±1.0)	48.1 (±0.9)	48.6 (±0.9)
MSD_11_	54.2 (±1.1)	58.1 (±0.9)	58.4 (±1.0)
MSD_15_	59.6 (±0.8)	63.6 (±0.6)	64.0 (±0.8)
MSD_19_	60.8 (±0.6)	65.9 (±0.5)	65.7 (±0.6)
SD of 270 (with sighs)			
SD	270 (±0)	474.4 (±5.4)	356.2 (±4.2)
LOESS_1_	269.7 (±0.2)	269.7 (±0.2)	276.1 (±1.1)
LOESS_0.75_	269.6 (±0.3)	269.6 (±0.3)	274.2 (±0.9)
LOESS_0.50_	269.6 (±0.3)	269.4 (±0.3)	272.1 (±0.8)
MSD_7_	234.2 (±3.1)	234.5 (±3.0)	235.2 (±3.1)
MSD_11_	259.5 (±3.2)	260.0 (±3.2)	261.0 (±3.2)
MSD_15_	272.1 (±2.6)	272.9 (±2.6)	273.7 (±2.7)
MSD_19_	273.6 (±2.1)	274.8 (±2.2)	275.4 (±2.2)
SD of 500 (with sighs)			
SD	500 (±0)	635.1 (±11.0)	552.8 (±7.3)
LOESS_1_	499.0 (±0.8)	499.0 (±0.8)	503.0 (±1.8)
LOESS_0.75_	498.6 (±1.0)	498.6 (±0.9)	501.4 (±1.7)
LOESS_0.50_	497.8 (±1.2)	497.8 (±1.2)	499.5 (±1.6)
MSD_7_	468.3 (±5.4)	468.5 (±5.3)	468.7 (±5.4)
MSD_11_	494.4 (±4.5)	494.7 (±4.5)	495.2 (±4.5)
MSD_15_	505.2 (±3.8)	505.6 (±3.9)	506.2 (±3.9)
MSD_19_	505.5 (±3.5)	506.1 (±3.6)	506.6 (±3.6)

*Note*: Simulations of VT with a true standard deviation of 60, 270, and 500 mL. The data were simulated with sighs.

Abbreviations: LOESS, locally estimated scatterplot smoothing with a span of n; MSD_n_, moving standard deviation using “n” data for the calculation of individual SD.

## 4 DISCUSSION

In these simulations, the LOESS method was highly precise and had a low bias. Trend (linear, exponential, or logarithmic) had little influences on the precision and bias of the method. LOESS_1_ gave overall the best results when there was no trend (flat) or a linear trend. The LOESS_0.5_ gave the best results for data with extreme trends (exponential or logarithmic). This was expected since a lower span is associated with a higher fit to the data when a trend is present but also associated with overfitting when no trend is present. Therefore, the LOESS_0.75_ might be a good trade‐off to describe both rest and exercise data during CPET. The bias and precision of the LOESS method were suboptimal in the simulations using extreme trends (logarithmic or exponential) combined with very low underlying dispersion. In these situations, the LOESS method resulted in an overestimation of the true value. For example, the LOESS_0.75_ for simulation of VT with a SD of 60 mL resulted in an average overestimation of the dispersion of 13.6 mL. The precision remained, however, very good (SD of 2.1 mL). This was also true for the simulations of sighs. These simulations were the only simulations where the MSD had a lower bias than LOESS. MSD in this specific scenario (small dispersion, extreme trends of VT) was slightly more precise for the VT simulations with no sighs and less precise for the VT simulations with sighs.

The MSD method was globally less precise and had higher bias than the LOESS method across the various simulations, except for the situation explained above (low dispersion and extreme trends). As expected, a small number of data used with the MSD (e.g., MSD_7_) usually resulted in an underestimation of the true parameter, while a high number of data used with the MSD (e.g., MSD_19_) usually resulted in an overestimation of the true SD parameter. Also, a lower number of data used with the MSD resulted in a lower precision of the measurement.

Applying a trend to the sighs did not perfectly represent all real‐life situations where the amplitude values of the VT spike might stay constant during exercise even though VT is progressively rising. Example of patients with frequent sighs are presented in Appendix S1 of our previously published case series (Genecand et al., 2023). In our simulations, we applied a trend to the sighs during exercise, therefore leading to an increase of the value of the sighs during exercise. While our simulations do not perfectly mimic real life, it mimics a more extreme situation where estimation might be more complicated. However, tested methodologies were still able to yield good estimates of the standard deviation with a small bias and a small dispersion and therefore should be able to measure the dispersion of data when sighs are present in real‐life situations.

Our study compared two methods for measuring data dispersion around breathing parameters during exercise to determine which is more accurate. Overall, the LOESS_0.75_ method appears effective in evaluating the dispersion of breathing parameters across most simulated scenarios and seems to be more precise with less bias than the MSD method in many situations. This approach could potentially describe the dispersion of various breathing parameters during different phases of cardiopulmonary exercise testing (CPET) with high precision and low bias.

**FIGURE 8:**
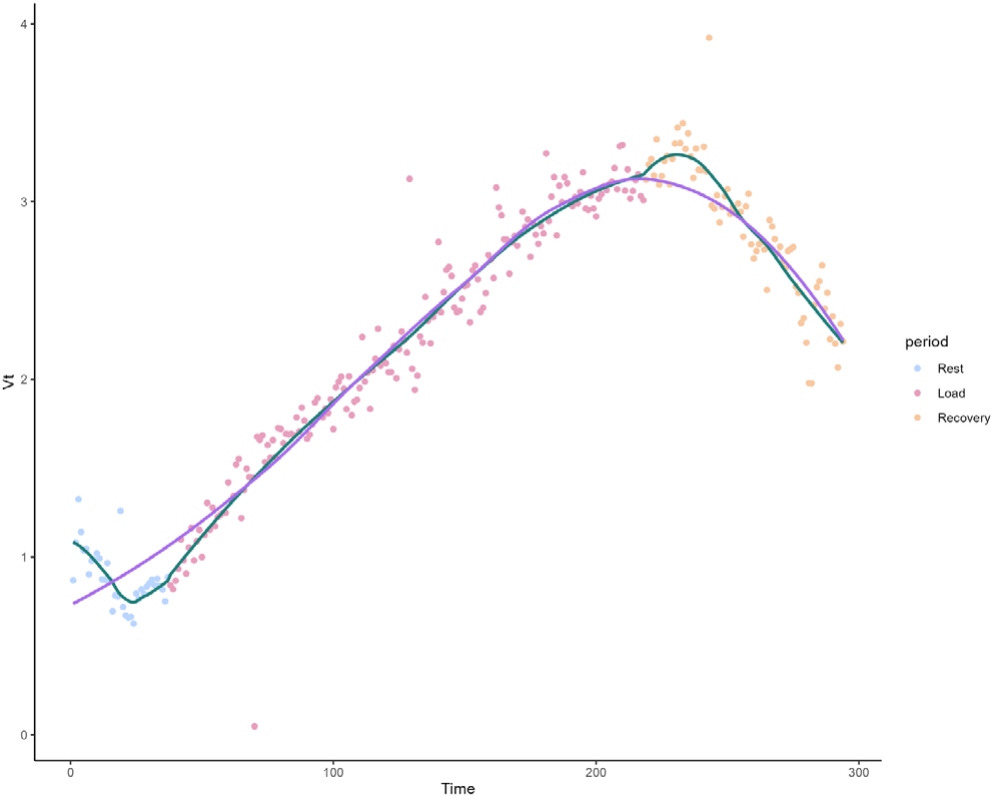
Example of the LOESS_0.75_ in a real patient. The regression using LOESS_0.75_ applied to the overall dataset is shown in purple. In green, the LOESS_0.75_ is calculated separately for each of the three periods: rest (blue points), exercise (red points), and recovery (yellow points). We observe that the fit improves when a separate regression is performed for each period, as the method struggles to adapt to extreme, rapid changes in trend, such as during the transition from rest to exercise, especially when a small number of points are contributing to these extreme changes. VT, tidal volume.

If the LOESS_0.75_ is used during CPET, we recommend applying a regression for each distinct exercise phase (rest, warm‐up, exercise, and cool‐down). Then the SD can be calculated on the overall residuals obtained with these LOESS_0.75_ or for each separate phases. This recommendation is due to the method's slower responsiveness to extreme and rapid changes in trend direction, especially when a small number of points are contributing to these changes. This is illustrated in Figure 8 from a real patient. We also recommend using a visual analysis of the regression slopes and the distribution of the residuals, which will help users of this method assess whether the fit of the LOESS 0.75 is appropriate for their data.

We believe the next steps in this research field should focus on determining whether the dispersion of breathing parameters is useful for diagnosing dysfunctional breathing, has prognostic value, and responds to interventions such as physiotherapy. Testing these methods on a large number of healthy patients could facilitate the development of normative values, which would be valuable for distinguishing between normal and abnormal dispersion of breathing parameters.

### 4.1 Other potential objective markers of DB

Some authors have attempted to describe data dispersion by using the difference between the highest and lowest 20‐second arithmetic means over the last full minute of rest, the second minute of unloaded exercise, and the third minute of loaded exercise (Mendes et al., 2023). Comparing 20 subjects with dyspnea (DB) to 10 healthy subjects, they demonstrated that this approach had some discriminative capability, with area under the curve (AUC) values ranging from 0.729 to 0.845. Although this method benefits from simplicity, it seems counterintuitive to derive breathing dispersion from average data rather than from the entire dataset.

Recently, Knöpfel et al. tested a log‐logistic regression model's ability to identify what clinicians consider high variability in tidal volume (VT) (i.e., DB with irregularity in the ventilatory response) (Knopfel et al., 2024). They achieved an AUC of 0.78 (95% CI 0.72, 0.83). To our knowledge, this is the first study to apply regression modeling to evaluate the dispersion of a breathing parameter (VT) in this context. However, their method assumes that the association between VT and VE follows a log‐logistic function which may not generalize to other parameters, such as breathing frequency (BF). This limitation contrasts with the non‐parametric methods we used, which can flexibly fit various breathing parameters.

Other objective methods may also be valuable in evaluating dysfunctional breathing (DB). Notably, entropy measurements, such as approximate entropy (ApEn) not corrected for trend, have demonstrated some ability to distinguish between healthy individuals and those with DB, making them undeniably interesting (Bansal et al., 2018; Samaranayake et al., 2023). Entropy measurements are used in time‐series analysis to assess data predictability (Pincus & Goldberger, 1994; Richman & Moorman, 2000). Interestingly, their calculation relies on the underlying data dispersion (i.e., the SD of the dataset) and was originally developed for trend‐free data (Pincus & Goldberger, 1994; Richman & Moorman, 2000). Thus, it is common practice to report dataset dispersion alongside entropy measurements (Pincus & Goldberger, 1994; Richman & Moorman, 2000). Our approach enables reporting of dispersion alongside entropy measurements, potentially enhancing the evaluation of DB in the future. A specific level of predictability is always interpreted within the context of a given dispersion, making these two concepts complementary. However, several questions remain before entropies can be widely adopted in clinical practice as objective markers of DB. For instance, how does the trend in breathing parameters impact entropy's discriminative ability? What are the effects of different calculation parameters, such as the coefficient for the tolerance interval (“r”) and the number of respiratory cycles (“N”)? Additionally, should we use approximate entropy or sample entropy?

### 4.2 Strength and limitations

The main strength of our study is the rigorous approach using simulations based on real‐life parameters to validate a method able to measure the dispersion of breathing parameters during exercise. When no gold standard exists, simulations are considered the most appropriate approach to validate new methodologies of measurement, and the inclusion of real‐life parameters allow these simulations to be used with confidence in real‐life setting (Morris et al., 2019).

The limitations of our study are the following: we based our simulations on real‐life data of patients with DB after COVID‐19 without cardio‐respiratory comorbidities and simulated the most extreme trends in our population. It seems reasonable to say that this method may be applicable to all populations that lie within the simulated parameters. However, it is possible that more extreme values, especially end values of BF and VT and extreme trends, might exist in other populations such as athletes or patients with other respiratory diseases. In these populations, we cannot assure that the tested methodologies will be as precise and accurate as demonstrated in the present study. Another limitation is that we only assessed two methods (LOESS and MSD) to estimate the dispersion of data and, therefore, other non‐tested methods might be more precise and accurate. Third, the measurement of the dispersion does not differentiate the reasons that lead to this dispersion. For example, methods will not differentiate sighs or purely erratic breathing when VT dispersion is measured. The cause of the dispersion should therefore ideally be further defined by the clinicians (pure dispersion of VT or sighs) before conclusions are drawn.

## 5 CONCLUSIONS

We demonstrated that the standard deviation of the residuals from a LOESS seems to be an appropriate method to measure the dispersion of both VT and BF at rest and during exercise. With a span of 0.75 and a 2nd degree polynomial, LOESS could be considered as the method of choice to quantify the dispersion of data both at rest and during exercise. MSD performed globally less well than LOESS except for extreme trends associated with a very low dispersion of data.

## AUTHOR CONTRIBUTIONS

L.G. and C.J conceived the study. C.J. designed and performed the statistical simulations. L.G. drafted the manuscript and handled the submission process. S.T. prepared some figures. P‐O.B., I.F., M.A., and L.G. analyzed the real‐life data of patients with post‐COVID‐19 DB that allowed for the statistical simulations. P‐OB, F.L, A.Ber, I.G, C.C, S.T, R.D, G.S. A.Bri, I.F, M.A, P.L, A.Beu, and D.M edited and re‐revised the first draft of the manuscript. All authors approved the final version of the manuscript.

## FUNDING INFORMATION

This study was supported by the Ligue pulmonaire genevoise, the Ligue pulmonaire valaisanne, the foundation Lancardis and the fondation Rankers Hartmann.

## CONFLICT OF INTEREST STATEMENT

PL reports personal fees and support for attending meetings and/or travel from Chiesi, AstraZeneca, and GSK, outside of this work. None of the other authors has any conflicts of interest, financial or otherwise, to disclose in relation with this work.

## ETHICS STATEMENT

The real‐life data were obtained from a previous study involving human participants, which was approved by the local ethics comittee commission cantonale d'étique de la recherche sur l'être humain (CER VD ID 2021‐01698). Participants gave written informed consent for participation in the research project.

## PATIENT AND PUBLIC INVOLVEMENT

Patients and/or the public were not involved in the design, or conduct, or reporting, or dissemination plans of this research.

## Supporting information


Appendix S1.
