# Supplementary material for: Comparing methods to measure the dispersion of breathing parameters during exercise testing: A simulation study based on real‐life parameters from patients with dysfunctional breathing
Source: Physiol Rep. 2025 Feb 28;13(5):e70233. doi: 10.14814/phy2.70233 (PMC11870078; doi:10.14814/phy2.70233)
Supplement: Supplementary file 1 — Appendix S1. [file PHY2-13-e70233-s001.docx]

Supplementary material – R script

1. **Simulations to assess the bias (underestimation) of the SD when using moving SD with different number of data points.**

### number of data points (3, 5, and 7)

moving_sd = function(x, nb_datapoint){

if ((nb_datapoint %% 2) == 0) stop('The number of datapoint must be odd (not even)')

temp = rep(NA, length(x))

nb_one_side = (nb_datapoint-1)/2

for (i in nb_one_side+1 : (length(x)-nb_one_side)){

temp[i] = sd(x[(i-nb_one_side) : (i+nb_one_side)])

}

return(mean(na.omit(temp)))

}

iterations = 10000

sd3 <- sd5 <- sd7 <- rep(NA, iterations)

set.seed(0)

for (i in 1:iterations){

x = rnorm(n, 0, 1)

sd3[i] = moving_sd(x, 3)

sd5[i] = moving_sd(x, 5)

sd7[i] = moving_sd(x, 7)

}

1-mean(sd3)

1-mean(sd5)

1-mean(sd7)

1. **Simulations for VT – R script**

moving_sd = function(x, nb_datapoint){

if ((nb_datapoint %% 2) == 0) stop('The number of datapoint must be odd (not even)')

temp = rep(NA, length(x))

nb_one_side = (nb_datapoint-1)/2

for (i in nb_one_side+1 : (length(x)-nb_one_side)){

temp[i] = sd(x[(i-nb_one_side) : (i+nb_one_side)])

}

return(mean(na.omit(temp)))

}

sd_loess_res = function(x, span=0.75){

mod = loess(x~time, df, span=span)

sd(mod$residuals)

}

# simulations -------------------------------------------------------------

n=300

# VT

min_value = 790

max_value = 2130

SD = 270 # 60 270 500

### Trends

# flat --------------------------------------------------------------------

flat = rep(min_value, n)

# linear ------------------------------------------------------------------

lineay = (1:n) / max(1:n) * (max_value-min_value) + min_value

# log ---------------------------------------------------------------------

logy = log((1:n)) / max(log((1:n))) * (max_value-min_value) + min_value

tendances = data.frame(flat, lineay, logy)

iterations=100

list_var = c('distr','sd','loess_100','loess_75','loess_50','msd_7','msd_11','msd_15','msd_19')

output = data.frame(matrix(ncol=length(list_var), nrow=iterations*length(tendances)))

names(output) = list_var

ligne = 1

set.seed(0)

for (i in 1:iterations){

for (tendance in names(tendances)){

temp_data = rnorm(n,0,1) ; temp_data = temp_data / sd(temp_data) * SD

x = temp_data

df = data.frame(x, time=1:length(x))

### sighs --> chunk adding sights. Comment out to ignore

soup = rnorm(n, 3500-min_value, 200)

delai = round(rnorm(n, 15, 3),0) ; delai = delai[delai>=3]

delai = cumsum(delai) ; delai = delai[delai<=300]

df$x[delai] = soup[delai]

df$x = df$x/sd(df$x) * SD

### end of sighs chunk

df$x = tendances[[tendance]] + df$x

x = df$x

output[ligne, ] =

c(tendance,

as.numeric(c(

sd(x),

sd_loess_res(x, span=1),

sd_loess_res(x, span=0.75),

sd_loess_res(x, span=0.5),

moving_sd(x, 7),

moving_sd(x, 11),

moving_sd(x, 15),

moving_sd(x, 19)

)))

ligne = ligne + 1

}

if (i%%100 == 0) print(i)

}

1. **Simulations for BF – R script**

moving_sd = function(x, nb_datapoint){

if ((nb_datapoint %% 2) == 0) stop('The number of datapoint must be odd (not even)')

temp = rep(NA, length(x))

nb_one_side = (nb_datapoint-1)/2

for (i in nb_one_side+1 : (length(x)-nb_one_side)){

temp[i] = sd(x[(i-nb_one_side) : (i+nb_one_side)])

}

return(mean(na.omit(temp)))

}

sd_loess_res = function(x, span=0.75){

mod = loess(x~time, df, span=span)

sd(mod$residuals)

}

# simulations -------------------------------------------------------------

n=300

# Breathing frequency

min_value = 18

max_value = 39

SD = 4 # 2 4 6

### Trends

# flat --------------------------------------------------------------------

flat = rep(min_value, n)

# linear ------------------------------------------------------------------

lineay = (1:n) / max(1:n) * (max_value-min_value) + min_value

# exp4 -------------------------------------------------------------------

exp4 = exp((1:n)/40) / max(exp((1:n)/40)) * (max_value-min_value) + min_value

tendances = data.frame(flat, lineay, exp4)

iterations=10000

list_var = c('distr','sd','loess_100','loess_75','loess_50','msd_7','msd_11','msd_15','msd_19')

output = data.frame(matrix(ncol=length(list_var), nrow=iterations*length(tendances)))

names(output) = list_var

ligne = 1

set.seed(0)

for (i in 1:iterations){

for (tendance in names(tendances)){

temp_data = rnorm(n,0,1) ; temp_data = temp_data / sd(temp_data) * SD

x = tendances[[tendance]] + temp_data

df = data.frame(x, time=1:length(x))

output[ligne, ] =

c(tendance,

as.numeric(c(

sd(x),

sd_loess_res(x, span=1),

sd_loess_res(x, span=0.75),

sd_loess_res(x, span=0.5),

moving_sd(x, 7),

moving_sd(x, 11),

moving_sd(x, 15),

moving_sd(x, 19)

)))

ligne = ligne + 1

}

if (i%%100 == 0) print(i)

}
